# Examining Associations Between Intimate Partner Violence-Related Brain Injury, Psychological Abuse, and Cognitive Functioning in Community Women

**DOI:** 10.1007/s10896-025-01032-7

**Published:** 2026-01-19

**Authors:** Aylin E. Tanriverdi, Grant L. Iverson, Eve M. Valera

**Affiliations:** 1 Department of Neurology, Massachusetts General Hospital, Boston, MA, USA; 2 Department of Psychiatry, Massachusetts General Hospital, Boston, MA, USA; 3 Department of Physical Medicine and Rehabilitation, Harvard Medical School, Boston, MA, USA; 4 Department of Physical Medicine and Rehabilitation, Spaulding Rehabilitation Hospital and the Schoen Adams Research Institute at Spaulding Rehabilitation, Charlestown, MA, USA; 5 Mass General for Children Sports Concussion Program, Boston, MA, USA; 6 Home Base, A Red Sox Foundation and Massachusetts General Hospital Program, Boston, MA, USA; 7 Department of Psychiatry, Harvard Medical School, 149 Thirteenth Street, Room 2660, Boston, Charlestown, MA 02129, USA

**Keywords:** Intimate partner violence, Brain injury, Psychological abuse, Reaction time, Memory, Cognitive functioning

## Abstract

**Purpose:**

Intimate partner violence (IPV) has been associated with adverse physical, psychological, and cognitive consequences in women. Long-term effects of IPV on cognitive functioning are not well understood. We studied cognitive functioning of community-residing women who experienced physical and psychological IPV, on average eight and five years prior, respectively. Specifically, we investigated cognitive functioning in relation to both number of IPV-related mild brain injuries (BIs) and psychological abuse severity.

**Methods:**

In a sample of 97 community dwelling women (age 19–69, mean = 39), we assessed Reaction Time, Verbal Memory, Visual Memory, Processing Speed, and Executive Function using the Central Nervous System Vital Signs computerized neuropsychological test battery; lifetime psychological abuse severity using a modified Revised Conflict Tactics Scale 2; and IPV and non-IPV related BIs using the Ohio State University Traumatic BI Identification Method and the BI Severity Assessment.

**Results:**

Women obtained lower scores than expected, given their level of education, on the Reaction Time, Verbal Memory, and the Executive Function domain scores, approximately one-third to two-thirds of a standard deviation below the normative means for the general population. 61% of our sample (59/97) reported at least one IPV-related BI. A greater number of IPV-related mild BIs was associated with lower Visual Memory scores, with a small effect size, but no other cognitive domain scores. Psychological abuse severity had a linear association with higher Reaction Time scores (indicating faster and more accurate responses). Psychological abuse severity had a quadratic inverted-U relationship with Verbal Memory scores indicating initially higher scores, but with greater abuse severity lower scores on the verbal memory test.

**Conclusions:**

Some of the women in the community who experienced physical and psychological IPV years ago obtained cognitive test scores that were lower than expected. There was a weak association between a greater number of IPV-related mild BIs and a measure of visual memory. Unexpectedly, we found that greater psychological abuse severity was associated with faster complex reaction time. Also, at high severity of psychological abuse, women tended to score worse on a measure of verbal recognition memory. These results should be interpreted with caution until replicated in other samples. Future studies using larger sample sizes and longitudinal study designs are needed to better understand the mechanisms by which experiencing psychological abuse is associated with future cognitive functioning.

## Introduction

Intimate partner violence (IPV), violence perpetrated against a partner, boyfriend, girlfriend, or spouse, is a prevalent problem afflicting 30% of women over age 15 worldwide (Devries et al., 2013). IPV has been linked to several physical, psychological, and cognitive consequences. Physical consequences include brain injuries (BIs), chronic disease, difficulty walking, and chronic pain, among others (Coker et al., 2000; Ellsberg et al., 2008; Valera & Berenbaum, 2003). Psychological impacts include distress, suicidality, posttraumatic stress disorder (PTSD), depression, and anxiety (Dokkedahl et al., 2022; Ellsberg et al., 2008; Nathanson et al., 2012; Valera & Berenbaum, 2003; Valera et al., 2022). IPV has also been linked to worse cognitive functioning. Compared to women who did not experience IPV, women who experienced IPV had lower scores on tests of autobiographical memory (Billoux et al., 2016), working memory (Fernández-Fillol et al., 2023; Stein et al., 2002), verbal episodic memory (Fernández-Fillol et al., 2023), visuoconstruction and visual memory (Stein et al., 2002), attention (Fernández-Fillol et al., 2023; Stein et al., 2002), and executive function (Seedat et al., 2005; Stein et al., 2002). Also, compared to control subjects who have not experienced IPV, women with IPV-related PTSD had obtained lower scores on tests of complex visuomotor processing speed (Aupperle et al., 2012), executive function (Aupperle et al., 2012, 2016; Stein et al., 2002), processing speed, and speeded fluency (Twamley et al., 2009).

### Relationship Between IPV-Related BI and Cognitive Functioning

Although several studies have examined the link between IPV and cognitive functioning, very few have focused on IPV-related BI. The nascent literature on IPV-related BI and cognitive functioning has suggested a negative relationship. For instance, IPV-related BI severity scores were negatively associated with measures of verbal learning (Valera & Berenbaum, 2003), long-term verbal memory (Quiroz-Molinares et al., 2023; Valera & Berenbaum, 2003), working memory (Quiroz-Molinares et al., 2023), and cognitive flexibility (Quiroz-Molinares et al., 2023; Valera & Berenbaum, 2003). In neuroimaging studies, IPV-related BI severity scores were negatively associated with functional (Valera & Kucyi, 2017) and structural neural connectivity (Valera et al., 2019). Functional connectivity in brain networks implicated in IPV-related BI were positively associated with verbal learning and memory (Valera & Kucyi, 2017). Women who had strangulation-related alterations in consciousness (AICs) obtained lower scores on a verbal memory test than women who did not have strangulation-related AICs (Valera et al., 2022). In a study testing cognitive-motor function, IPV-related BI severity scores were associated with fewer number of targets hit, fewer number of objects (targets and distractors) hit, and slower hand speed (Maldonado-Rodriguez et al., 2021). Many of these associations were found in samples of mostly shelter dwelling women and those experiencing recent IPV. As such, studies on women currently residing in a shelter are likely to capture short-term effects of IPV-related BIs. For example, in Quiroz-Molinares et al. (2023)’s sample, the time since the last abuse ranged from less than 1 month to 12 months.

To better understand the possible longer-term effects of IPV-related BI on women who are no longer in immediate crisis, we used a sample of community dwelling women, i.e., women living at home, not in a shelter. Their last physical abuse incident occurred eight years ago on average, whereas their last IPV-related mild BI occurred 10 years ago, on average. Most studies of IPV-related BI have been done with women who are residing in shelters or otherwise seeking help for IPV. As such, it is unclear whether observed associations with IPV-related BI would persist after the acute traumatic aspects of IPV were removed. Examining the long-term correlates of IPV-related BI is important for understanding the health problems and treatment needs of these women. We focused specifically on mild IPV-related BI because previous research has shown that most IPV-related BIs are classified as mild (Quiroz-Molinares et al., 2023; Valera & Berenbaum, 2003) and are often repetitive (Valera & Berenbaum, 2003). Previous studies on repetitive mild BI have been primarily conducted with male athletes or military veterans. Their results may not generalize to women who experience IPV whose injuries are often unrecognized and untreated, among other abuse-related barriers to recovery (Murray et al., 2016; Valera et al., 2019a). Although the literature on repetitive BI in IPV remains limited, one study found that among women who experienced IPV, a greater cumulative history of head injuries was associated with more severe physical symptoms such as headaches, dizziness, and sleep problems (Karr et al., 2024). Building on this work, the present study aimed to explore the longer-term cognitive outcomes in a sample of community dwelling women who had a history of mild repetitive IPV-related BIs.

### Relationship Between Psychological IPV and Cognitive Functioning

It is common for women experiencing IPV to experience multiple types of abuse from their partners (Krebs et al., 2011). In particular, studies have found that women who experience physical abuse tend to also experience psychological abuse (Krebs et al., 2011). Several studies have found a link between psychological IPV and worse mental health (Beeble et al., 2009; Blasco-Ros et al., 2010; Coker et al., 2002; Começanha et al., 2017; Estefan et al., 2016; Pico-Alfonso, 2005). However, there is a dearth of research on the relationship between psychological abuse and cognitive function. One study by Stein et al. (2002) did not identify significant correlations between psychological abuse severity and a range of neuropsychological measures, but they also did not report controlling for other variables. Daugherty et al. (2019) investigated the relationship between physical IPV, psychological IPV, and cognitive functioning. Compared to no IPV controls, women with simultaneous physical and psychological IPV had lower scores on visuomotor skills, attention, inhibition, planning, reasoning, and decision-making, and women with only psychological IPV had lower scores on attention and decision making. Compared to women with psychological IPV only, women with simultaneous physical and psychological IPV had lower cognitive flexibility scores (Daugherty et al., 2019).

These findings suggest that the cognitive test scores may be worse for women who have experienced both physical and psychological IPV. However, little is known about the differential effects of IPV-related BI and psychological IPV on women’s cognitive function. The aforementioned IPV-related BI studies did not account for the specific effects of psychological IPV (though abuse severity more generally was considered), and the psychological IPV studies did not characterize the potential presence of mild BIs. Stein et al. (2002) excluded people with “serious” head injuries, i.e., loss of consciousness (LOC) greater than ten minutes, and Daugherty et al. (2019) reported excluding participants with traumatic BIs. To address these gaps, we have investigated IPV-related mild BIs and psychological abuse severity in relation to cognitive functioning in women who experienced IPV.

### Study Aims and Hypotheses

As the foregoing literature review indicates, the nascent literature on the associations between IPV-related BIs and cognitive functioning has not accounted for the role of psychological abuse. Likewise, the burgeoning literature on the associations between psychological abuse and cognitive functioning has not controlled for the role of IPV-related BIs. Thus, we do not know if the associations with cognitive functioning are more strongly associated with IPV-related BIs or psychological abuse, or both. We aim to address this gap. In this study, we have conducted a focused exploratory analysis to investigate how cognitive functioning is associated with IPV-related brain injuries (BIs) and psychological abuse. We have hypothesized that higher levels of both IPV-related mild BIs and psychological abuse will have distinct associations with worse cognitive functioning.

## Methods

### Sample and Data

Data collection took place between 2020 and 2023 as part of a larger, ongoing study on the effects of IPV-related BI. Participants were recruited through flyers shared with community partners, the institution’s online research platform, and social media. Community partners included hospital-based programs, police department-affiliated initiatives, and community-based organizations focused on violence prevention and survivor support. To be included, women needed to have experienced at least one instance of physical partner violence ever and be at least 18 years old. A personal history of brain injury was not required for eligibility.

Participants in the current study were drawn from the larger study but met additional exclusion criteria. Specifically, women were excluded if they were currently living in a shelter, had sustained any BI within the past three months, had a history of moderate to severe brain injury, or had a neurological condition (e.g., epilepsy). We wanted to study possible long-term effects of or associations with repetitive mild BI so did not include women who might be experiencing acute or subacute effects of injury. After applying all exclusion criteria, all participants in the final sample who reported a BI had sustained it more than one year prior to study participation. At the time the analysis was done, our sample consisted of 124 women. Table 1 presents the sample construction process, and the number of subjects dropped due to not meeting inclusion criteria of the study.

#### Remote Data Collection Procedures

This study was conducted remotely via video conference (Zoom) in a single, 2–3 h session. Participants were instructed to find a location where they could be distraction-free and comfortable for the duration of the session. To accommodate participants without access to a personal computer, we offered the option to use a study computer at the research center, where a study staff member, located in a room separate from the participant, would host the assessment via video conference. Following informed consent, demographic information was collected via interview. Participants then completed the computerized CNS Vital Signs (CNS-VS) cognitive battery, which was designed to be completed independently. Each participant received an access code and logged into the CNS-VS platform, where they followed standardized, on-screen instructions. Upon completion, participants notified the interviewer, who then resumed the session. Participants were sent secure links via REDCap to complete surveys about symptoms of depression, anxiety, and traumatic stress, as well as alcohol use, partner violence, and childhood abuse. Finally, information about general abuse history and brain injury history were obtained via interview. No participants in this sample reported being in a physically abusive relationship at the time of participation. Nevertheless, study staff offered participants the opportunity to create a safety plan in advance, which included strategies for discreetly pausing or redirecting the session if needed. Participants were reminded that they could take breaks or withdraw from the study at any time. Each participant received a $50 gift card for completing the study session. All processes and procedures were approved by our hospital’s Institutional Review Board (IRB).

### Measures

#### Cognitive Functioning

We assessed cognitive functioning using *Central Nervous System Vital Signs* (CNS-VS), a standardized computerized neuropsychological test battery (Gualtieri & Johnson, 2006). We focused on single test domains that measure specific cognitive functions, such as complex reaction time (embedded in a Stroop task), verbal recognition memory for words, visual recognition memory for designs, processing speed, and fluid executive functioning, because previous work discussed above suggests that IPV may be associated with these cognitive functions. We used the embedded Validity Indicator (VI) to identify and exclude invalid test scores. The proportion of invalid test scores were as follows: 1/97 for Reaction Time, 0/97 for Verbal and Visual Memory, 1/97 for Processing Speed, and 5/97 for Executive Function. Participants completed the CNS-VS assessment immediately after being consented, prior to abuse history and BI interviews, to minimize bias. We used participants’ standard scores in the analysis, which are automatically calculated by CNS-VS based on an age-normalized sample. Higher scores indicate better performance. We accounted for outlier standard scores by truncating scores that were 4 standard deviations (SD = 15) above and below the population mean (100). The domain scores and tests are described in Table 2 below.

#### Brain Injuries

We used the mild BI definition developed by the American Congress of Rehabilitation Medicine Special Interest Group on Mild TBI: “a traumatically induced physiological disruption of brain function, as manifested by at least one of the following: any loss of consciousness (LOC); any loss of memory for events immediately before or after the accident; any alteration in mental state at the time of the accident (e.g., feeling dazed, disoriented, or confused); focal neurologic deficit(s) that may or may not be transient” (Head Injury Interdisciplinary Special Interest Group of the American Congress of Rehabilitation, 1993). Following this definition, alterations in consciousness (AICs) resulting from violent shaking and strangulation-induced anoxia or hypoxia were also classified as BIs, consistent with previous research (Valera & Berenbaum, 2003; Valera et al., 2022). BIs were considered mild if AIC/LOC was less than or equal to 30 min, or if post-traumatic amnesia was less than or equal to 24 h. We defined “repetitive” BI as more than one BI.

We assessed the total number of BIs sustained across the lifespan using the *Ohio State University (OSU) Traumatic Brain Injury (TBI) Identification (ID) Method* and the *Brain Injury Severity Assessment (BISA)*. The OSU-TBI-ID is a standardized, validated method for obtaining the lifetime history of TBI through a 3–5 min structured interview (Corrigan & Bogner, 2007). The BISA is a semi-structured interview used to assess partner and non-partner related AICs in women who have experienced IPV, including strangulation-related AICs and TBIs (Valera & Berenbaum, 2003). The BISA has been widely used in IPV-related BI research (Adhikari et al., 2024; Macaranas et al., 2025; Maldonado-Rodriguez et al., 2021; Quiroz-Molinares et al., 2023; Saadi et al., 2022; Smirl et al., 2019; Valera & Kucyi, 2017; Valera & Berenbaum, 2003; Valera et al., 2019a b, 2022; Wallace et al., 2024). AICs (e.g., “blacking out”, feeling dizzy or stunned, seeing stars or spots, or experiencing a memory gap) served as indicators of brain injury. If a participant reported an AIC following any incident with a partner, follow-up questions were asked to identify the mechanism of injury (e.g., strangulation or blunt force to the head). If an AIC resulted from strangulation or choking, we classified it as a “strangulation-AIC,” which may include an anoxic, hypoxic, or ischemic brain injury. If an AIC resulted from traumatic forces to the head, it was classified as a TBI. This approach ensured that we identified strangulation and blunt force trauma that resulted in an AIC, which we considered a mild brain injury. If there was a strangulation event that did not result in an AIC, it was not recorded. This is consistent with how strangulation-AICs and TBIs were classified in prior work (Joseph Denk et al., 2025; Valera & Berenbaum, 2003; Valera et al., 2022). Data from both the OSU-TBI-ID and BISA were used to determine the number of IPV and non-IPV related BIs. The number of BIs were capped at 25, beyond which it was difficult to obtain an accurate estimate. We also used the number of non-IPV related mild brain injuries as a covariate.

#### Lifetime Partner Psychological Abuse and Negotiation

We assessed lifetime partner psychological abuse and negotiation with the *Revised Conflict Tactics Scale* (CTS2) (Straus et al., 1996) because it is widely used for measuring IPV. Although the original version assesses abuse within the past year, the developers note that the reference period can be adapted based on study aims. Because our focus was on the possible long-term correlates and effects of IPV and several participants had not experienced abuse in the past year, we modified the CTS2 to assess experiences both within and before the past year. Limiting the timeframe would have excluded important histories of violence that were essential for understanding the broader context of participants’ experiences. We note that the CTS2 has not been formally validated for lifetime IPV assessment, and we consider this a limitation.

We assessed psychological abuse using the psychological aggression subscale of the CTS2 which asked about verbally and emotionally abusive incidents such as insults, physical threats, and anger (e.g., a partner insulted or swore at you, destroyed something belonging to you). We included an additional item from the Severity of Violence Against Women Scale (SVAWS) (Marshall, 1992): i.e., “A partner threatened to kill you with a club-like object” to capture severe forms of psychological abuse that may not be fully reflected by the CTS2 alone, consistent with Valera and Berenbaum (2003). While the CTS2 is widely used to assess both aggressive and non-aggressive conflict behaviors within marital, cohabiting, or dating relationships, the SVAWS was developed to assess the severity of violence specifically in the context of intimate relationships. We felt it was important to include an item that reflected the intensity of participants’ lived experiences, particularly forms of psychological aggression that are highly threatening. We note that adding an additional item to a scale might influence its interpretability, and possibly aspects of reliability and validity.

Because some participants had been in multiple relationships, including supportive ones, we also controlled for partner negotiation in our analyses. To measure partner negotiation, we used the negotiation subscale of CTS2, which measured how effectively partners managed to communicate and resolve disagreements, including showing care despite disagreements, explaining one’s side, respecting feelings, believing in solving problems together, suggesting compromises, and agreeing to try solutions.

To score the measure, we followed the guidelines outlined in the CTS2 handbook. Participants reported how often each behavior occurred with their partner, either in the past year or before the past year. Each response option was assigned a midpoint value to approximate frequency: never (0), once (1), twice (2), 3–5 times (4), 6–10 times (8), 11–20 times (15), and more than 20 times (25). We calculated separate subscale scores (e.g., psychological aggression) by summing the midpoint values for all relevant items within each time period. We summed the past year and before past year scores to create lifetime scores. Higher scores reflect greater exposure to these behaviors.

#### Alcohol and Drug Use

Alcohol use in the past year was assessed using the *Alcohol Use Disorders Identification Test (AUDIT*) (World Health Organization et al., 2001) a widely used 10-item screening test (Bradley et al., 2003). Each AUDIT item is scored from 0 to 4. Total scores are calculated by summing across all items, yielding a possible range from 0 to 40. Higher scores reflect greater hazardous and harmful drinking. The AUDIT total score was used as a covariate in the analyses.

Chronic drug use was assessed via telephone interview by trained study staff. Participants were asked about their current use and times of heaviest use of drugs, the type of drug, when they started and stopped using the drug(s), the frequency of use, and whether their use led to problems in their lives. The presence of chronic drug use was determined by the study principal investigator. If participants described extended periods of consistent use, rather than occasional or one-time experimentation, and reported significant problems (such as addiction, loss of control, job loss, legal issues, or disrupted relationships), they were classified as chronic users. Although no strict numerical threshold was applied in advance, those who met our criteria reported using substances at least five days per week for a minimum of two years. Importantly, none of the participants identified as chronic users were actively using substances at the time of the study, reducing the likelihood that acute effects of substance use influenced study outcomes. A dichotomous variable for chronic drug use history was used as a covariate in the analysis (1: chronic drug use history, 0: no chronic drug use history). Participants were also asked whether they had ever taken medications for emotional or psychological reasons. A dichotomous variable for current psychotropic medication use was used as a covariate (1: currently on psychotropics, 0: not currently taking).

#### Childhood Trauma

Childhood abuse and neglect was assessed using the 34-item version of the Childhood Trauma Questionnaire (Bernstein et al., 1994), a validated questionnaire (Bernstein et al., 1997). The CTQ asked participants to report how frequently they experienced undesirable incidents in childhood, including physical, sexual, and emotional abuse, as well as physical and emotional neglect. Responses are rated on a 5-point Likert scale ranging from 1 (never true) to 5 (very often true), with some items reverse scored. Scores are summed to produce a total score, with possible values ranging from 25 to 125. Higher CTQ scores indicate greater severity of childhood abuse and neglect.

#### Psychological Distress

Symptoms of depression were assessed with the widely used Patient Health Questionnaire-9 (PHQ-9) (Kroenke et al., 2001). Each of the 9 items is rated on a 0–3 scale based on symptom frequency from “not at all” to “nearly every day,” yielding a total score ranging from 0 to 27. Higher scores indicate greater depressive symptom severity. Anxiety symptoms were assessed with the widely used Generalized Anxiety Disorder-7 (GAD-7) (Löwe et al., 2008; Spitzer et al., 2006). Each of the 7 items is scored on a 0–3 scale, based on symptom frequency from “not at all” to “nearly every day.” Total scores range from 0 to 21, with higher scores indicating greater severity of anxiety symptoms. Traumatic stress symptoms were assessed with the widely used PTSD Checklist-5 (PCL-5) (Blevins et al., 2015; Weathers et al., 2013). Participants rated the severity of 20 symptoms on a 5-point Likert scale ranging from 0 (not at all) to 4 (extremely). Total scores range from 0 to 80, with higher scores indicating greater severity of PTSD symptoms. Each score was used as a covariate in the analyses.

#### Abuse Recency and Demographics

Abuse recency and demographic variables (years of education, employment status, ethnicity/race) were obtained by trained study staff in a video interview. Years of education and employment status (1: employed, 0: not employed) were used as covariates in the analysis.

### Data Analysis

Bivariate Pearson correlations were used to examine the unadjusted associations between the number of IPV-related BIs and the neuropsychological domain scores. One-sample t-tests were conducted to assess whether the sample means in each neuropsychological domain significantly differed from the age-normalized population mean. We analyzed the data using ordinary least squares (OLS) regressions. Due to listwise deletion of missing observations across all study variables, the retained sample size drops from *N* = 97 to *N* = 70 to *N* = 76 in different regressions. We used scores on five tests of cognitive functioning as our dependent variables: i.e., Reaction Time, Verbal Memory, Visual Memory, Processing Speed, and Executive Function. The main independent variables of interest were the number of IPV-related mild BIs and psychological abuse severity.

Other factors such as alcohol and drug use, mental health difficulties, psychoactive medications, and adverse childhood experiences could also be associated with differences in cognitive functioning over and beyond the associations explained by IPV-related BIs and psychological abuse (Esopenko et al., 2024; Maldonado-Rodriguez et al., 2021). However, some previous studies on IPV-related BIs or psychological abuse excluded from their samples women with alcohol or drug use history (Daugherty et al., 2019; Valera & Berenbaum, 2003; Valera et al., 2022), women with significant mental health problems, and women taking psychoactive medications (Daugherty et al., 2019). Some did not account for childhood abuse (Daugherty et al., 2019; Valera & Berenbaum, 2003). We did not exclude these women in our sample to allow for a more representative sample. Instead, we attempted to adjust for these factors and others that were previously shown to be associated with our dependent variables: number of non-IPV related mild BIs, degree of partner negotiation, degree of alcohol use in the past year, presence or absence of chronic drug use history, current psychotropic medication use, degree of childhood trauma, traumatic stress symptom severity, depression symptom severity, anxiety symptom severity, years since psychological and physical abuse, years of education, and employment status. Instead of years since BI, we chose to control for years since physical abuse, a closely correlated proxy (*r* =.70, *p* <.01), to preserve sample size and avoid listwise deletion of participants without a BI history.

We also conducted a sensitivity analysis using a binary classification of IPV-BI (yes/no). Linear regression models were run adjusting for key demographic variables (age, education, and employment status), with cognitive functioning domains as the dependent variables. Further, we tested whether time since the most recent IPV-related BI influenced the association between the number of BIs and cognitive functioning.

Skewness and kurtosis values in Table 3 were in the normal range of variable distributions after log transformation of three skewed variables. Visual inspection of Q-Q (quantile-quantile) and residual plots also indicated that the normality of errors and constant variance of errors assumptions were satisfied. A visual inspection of the scatterplot of Verbal Memory versus psychological abuse severity and the residual plot of the Verbal Memory regression model showed that the data did not follow a linear pattern but rather an inverted-U (quadratic) pattern. Thus, we included a quadratic term of psychological abuse severity, defined as “CTS2 squared.”

## Results

In our sample, 61% of women reported at least one IPV-related mild BI. Of these women, 58% reported *more* than one IPV-related mild BI. As Table 3 shows, the cognitive domain scores were as follows: Reaction Time (Mean = 90.53, SD = 15.21), Verbal Memory (Mean = 94.70, SD = 18.14), Visual Memory (Mean = 96.86, SD = 15.32), Processing Speed (Mean = 99.32, SD = 13.63), and Executive Function (Mean = 93.59, SD = 14.74). For reference, the age-normalized mean for the general population in each domain is 100 with a standard deviation of 15. The differences from the population mean were statistically significant for Reaction Time (t(93)= −6.04, *p* <.001), Verbal Memory, (t(92)= −2.82, *p* =.006), and Executive Function, (t(90)= −4.15, *p* <.001), with medium to small effect sizes (Cohen’s d= −0.62, −0.29, and − 0.43, respectively).

Bivariate Pearson correlations were used to examine the unadjusted associations between the number of IPV-related brain injuries and the neuropsychological domain scores. The correlations between number of IPV-related brain injuries and the domain scores were as follows: Reaction Time *r* = −.17 (*p* =.112), Verbal Memory *r* = −.02 (*p* =.866), Visual Memory *r* = −.28 (*p* =.006), Processing Speed *r* = −.13 (*p* =.215), and Executive Function *r* = −.04 (*p* =.689). There was a small significant negative linear association between the number of IPV-related mild BIs and Visual Memory scores. There were no other bivariate associations between IPV-related mild BIs and cognitive domain scores.

The *Pearson correlations* between psychological abuse severity and the neuropsychological domain scores were as follows: Reaction Time *r* =.39 (*p* <.001), Verbal Memory *r* =.08 (*p* =.465), Visual Memory *r* =.012 (*p* =.916), Processing Speed *r* =.12 (*p* =.321), and Executive Function *r* =.18 (*p* =.129). There was a small-medium association between psychological abuse severity and Reaction Time scores. There were no other bivariate associations between psychological abuse severity and cognitive domain scores.

**Fig. 1:**
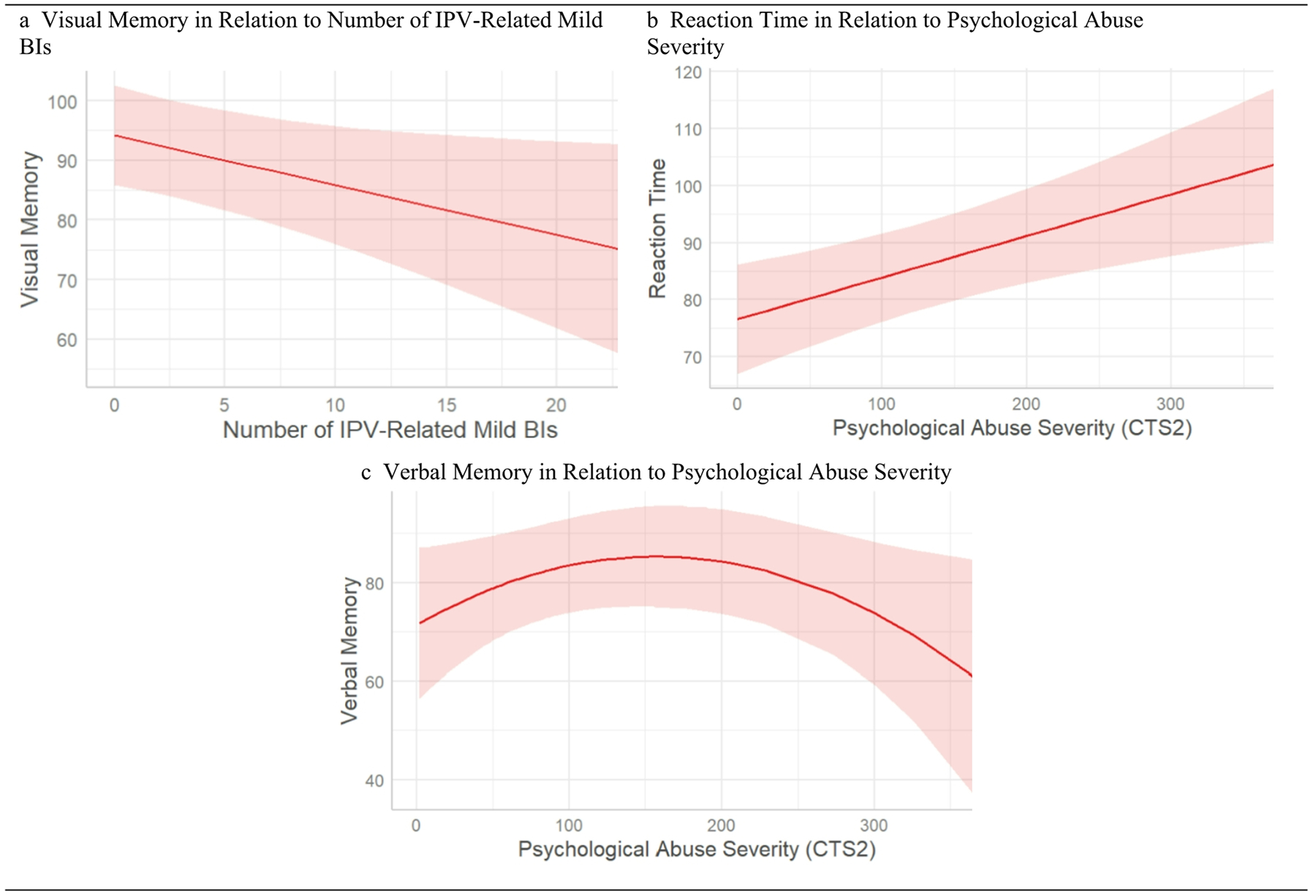
Plots of Regression Models **a**. Number of IPV-related mild BIs had a negative linear association with Visual Memory. **b**. Psychological abuse severity had a positive linear association with Reaction Time. **c**. Psychological abuse severity had a negative quadratic association with Verbal Memory. Lower cognitive test scores indicate worse performance. 95% confidence intervals are shaded.

Table 4 presents the *regression* results and Fig. 1 presents plots of the significant associations. For all of the cognitive domain scores, higher scores indicate better performance. As shown in Model-3 and Fig. 1a, number of IPV-related mild BIs had a negative linear association with Visual Memory (beta= −0.83, *p* =.033). We did not find statistically significant associations between the number of IPV-related mild BIs and Reaction Time, Verbal Memory, Processing Speed, or Executive Function.

The IPV-BI (yes/no) group-based analyses revealed no statistically significant differences in cognitive test scores between women with a history of at least one IPV-BI and those with no history of IPV-BI (*p*-values for IPV-BI variable in regression models for respective cognitive variables: Reaction Time: *p* =.64, Verbal Memory: *p* =.61, Visual Memory: *p* =.15, Processing Speed: *p* =.98, Executive Function: *p* =.50). The interaction between time since last IPV-related BI and number of BIs for predicting neuropsychological outcomes was not statistically significant (*p*-values for interaction term in regression models for respective cognitive variables: Reaction Time: *p* =.46, Verbal Memory: *p* =.36, Visual Memory: *p* =.98; Processing Speed: *p* =.35; Executive Function: *p* =.46).

As shown in Model-1 and Fig. 1b, psychological abuse severity had a positive linear association with Reaction Time (beta = 0.07, *p* =.002). As shown in Model-2, the quadratic term of psychological abuse severity (CTS2 squared) had a negative association with Verbal Memory (beta= −0.001, *p* =.039), indicative of a quadratic, inverted-U relationship, illustrated in Fig. 1c. This last result suggests that, all else being equal, low psychological abuse severity was associated with higher Verbal Memory scores, but moderate to high psychological abuse severity was associated with lower Verbal Memory scores. We did not find statistically significant associations between psychological abuse severity and scores on Visual Memory, Processing Speed, or Executive Function.

## Discussion

In this study, we sought to determine whether a past history of IPV-related brain injuries and psychological abuse would be associated with current cognitive functioning. More than half of this sample of women reported more than one IPV-related brain injury. We first note that, on average, our sample of women had lower scores than expected on the Reaction Time, Verbal Memory, and Executive Function domain scores. The average level of education for this sample was 15.8 years, just less than a college degree. Yet their mean normative scores for the Reaction Time, Verbal Memory, and Executive Function domain scores were 90.5, 94.7, and 93.6, respectively, which are roughly one-third to two-thirds of a standard deviation below the normative means for the general population (i.e., normative mean = 100, SD = 15). There was a small association between more IPV-related mild BIs and lower scores on the Visual Memory domain scores. However, no other cognitive domain score was associated with IPV-related BIs. Unexpectedly, we found that greater psychological abuse severity was associated with higher Reaction Time domain scores (indicating faster responses and overall better performance). Additionally, psychological abuse severity had a quadratic inverted-U relationship with Verbal Memory domain scores. At high severity of psychological abuse, women tended to score worse on this measure of verbal recognition memory.

Previous IPV literature has collectively identified a variety of factors that are associated with cognitive functioning. However, individual studies examined the associations with one or a few factors at a time, in isolation of the others. This has limited our understanding of the relative effects of the various factors on cognitive functioning. Our study aimed to address this limitation by including several determinants and examining their associations with cognitive functioning simultaneously. We discuss our findings and potential interpretations here.

### IPV-related Mild BI and Cognitive Functioning

We found that the number of IPV-related mild BIs was linearly associated with lower scores on a test of visual recognition memory. It is worth noting that it had been eight years, on average, since these women experienced physical abuse. Among those that had IPV-related mild BIs, it had been 10 years, on average, since the last IPV-related mild BI. Additionally, all IPV-related mild BIs occurred at least a year prior to the cognitive testing. Our results indicate that a negative association between IPV-related mild BI and visual memory is not limited to women living in shelters or those currently in crisis.

Previous studies have examined associations between IPV and visual memory, but they did not study IPV-related BIs per se. The findings from previous studies are mixed. For instance, Stein et al. (2002) found lower scores on visual memory in women with physical and/or sexual IPV than women without IPV. Twamley et al. (2009) found no difference in visual memory between an IPV-related PTSD group and no IPV/PTSD group. These studies excluded people with “serious” or moderate to severe brain injuries, i.e., loss of consciousness (LOC) greater than ten minutes, and they did not characterize the potential presence of mild BIs. It is possible that IPV-related mild BIs may be contributing to the inconsistencies in prior studies. We found an independent association between IPV-related mild BIs and visual memory, after controlling for several determinants of cognitive functioning, in response to calls to differentiate the effects of IPV-related BI from other forms of trauma and psychopathologies (Esopenko et al., 2024; Maldonado-Rodriguez et al., 2021). That said, the association with visual memory in our study was somewhat small. Notably, adults who sustain an mTBI are not expected to have persisting cognitive problems after 1–3 months post injury, as measured by neuropsychological tests (Karr et al., 2014; Rohling et al., 2011). Moreover, adolescents with a history of multiple past concussions do not obtain lower scores on neuropsychological tests than adolescents who have no prior concussions (Brooks et al., 2016, 2018; Iverson et al., 2019, 2021). Therefore, it will be important to determine if the present finding replicates. Future studies that examine the relationship between visual memory performance and everyday functioning could help determine the practical and clinical significance of this finding.

We did not find that the number of IPV-related mild BIs were significantly associated with verbal recognition memory or a measure of fluid executive functioning (set shifting). Prior studies reported that the IPV-related BI severity score was negatively associated with verbal memory and cognitive flexibility (Quiroz-Molinares et al., 2023; Valera & Berenbaum, 2003). Possible reasons for this difference may include the use of different cognitive tests under different conditions, as well as differences in sample characteristics, such as the time since IPV and/or time since most recent brain injury. For example, the average woman in our sample had several more years between their last IPV incident and cognitive testing, compared to the sample from Quiroz-Molinares et al. (2023) who experienced IPV within the past year of cognitive testing. Further studies with larger sample sizes might help clarify the mixed findings to date.

The absence of significant differences in cognitive test scores between women with at least one IPV-BI and those with no IPV-BI history suggests that group-based analyses may not be sensitive enough to capture these differences, if present. In contrast, a continuous IPV-BI measure may preserve meaningful variability and identify associations that could be obscured by binary categorization.

### Psychological Abuse and Cognitive Functioning

Psychological abuse severity was not associated with visual recognition memory, processing speed, or fluid executive functioning. Compared to women who reported less severe psychological abuse, women who reported more severe psychological abuse tended to score higher on a complex reaction time test, where higher scores indicated faster and more accurate responses.

Verbal recognition memory test scores showed a quadratic inverted-U relationship with psychological abuse severity. While zero to low levels of psychological abuse were associated with slightly higher verbal recognition memory scores, high levels of psychological abuse were associated with lower verbal recognition scores. The unusual association that we found between verbal recognition memory test scores and psychological abuse severity is small and its practical and clinical significance were not assessed within this study. That said, a prior study also reported quadratic inverted-U associations. Zuelsdorff et al. (2023) found that the number of traumatic events experienced during adulthood had a quadratic inverted-U relationship with measures of global cognition, immediate verbal recall, and delayed verbal recall in a cross-sectional analysis. The authors suggested that if traumatic events were rare, they may “require adaptation that fosters personal resilience,” but higher exposures to traumatic events could be associated with “significant cognitive detriments” (Zuelsdorff et al., 2023).

Future research may examine factors such as potentially protective cognitive adaptations (e.g., using one’s “problem-solving brain” to protect oneself from the abuser) (Boxall, 2023), therapeutic and social support (Lelaurain et al., 2017), healthy coping behaviors (Logemann-Molnár et al., 2024; Lueke & Lueke, 2019; Oberste et al., 2019), resilience (Yıldız-Akyol & Öztemel, 2024), and post-traumatic growth (Kira et al., 2022) to increase our understanding and interpretation of these findings.

### Limitations and Future Work

This study has several limitations. First, like most IPV studies, it used a cross-sectional research design, which does not allow causal inference. Second, all data were self-reported and could not be verified with other sources. Third, the cognitive testing was conducted remotely, over videoconferencing, not in person. Fourth, listwise-deletion of missing observations reduced the sample size and statistical power in regression analyses. Despite this limitation, our study revealed several significant relationships. However, the limited sample size constrained our ability to examine the distinct cognitive effects of IPV-related mild TBIs versus strangulation-related AICs. Future studies with larger sample sizes and greater statistical power are needed to replicate or refute the current findings and to more precisely evaluate the associations with specific mechanisms of brain injury. Fifth, our study did not have a comparison group without any history of physical partner violence. Future studies should include such a group to better understand the distinct effects of psychological abuse. We also note as a limitation that chronic drug use classification was based on investigator judgment rather than a validated assessment. Finally, while we tested whether the passage of time influenced the association between the number of BIs and cognitive functioning, this study did not account for other potentially important confounding or moderating variables, such as cognitive recovery, that could be related to outcomes. Future studies could also include measures of women’s resilience, help seeking behavior, access to support resources/growth opportunities, type of therapy received, coping behaviors adopted, level of social support, and the extent to which women may have developed adaptive cognitive abilities.

### Implications for Research and Clinical Practice

We found statistically significant associations between some cognitive test scores and several of the control variables included in the regression model, including partner negotiation, chronic drug use history, current psychotropic use, childhood trauma, PTSD symptomology, and depression symptomology (see Table 4). Future research should study these factors and variables when investigating associations between IPV and cognitive functioning. For example, because traumatic stress is both a potential outcome of IPV and a factor known to impact cognitive functioning, future research should examine traumatic stress as a primary variable of interest, rather than solely as a covariate, to better understand its independent and interactive effects with brain injury on cognitive outcomes. We also observed null or small-effect associations between the number of IPV-related brain injuries, psychological abuse, and cognitive functioning. One possible interpretation is that some women may experience cognitive recovery over time following IPV-related BI and psychological abuse. Future research should be designed to examine whether time since injury or cognitive recovery might mediate or influence associations or interactions among IPV-related brain injuries, psychological abuse, and cognitive functioning. It would also be important to examine what types of treatment services women received and whether they were deemed helpful. A key aspect of our study was its community-based sample, i.e., women were not in shelters and they may or may not have been connected with IPV support services or other health care services. Studies are needed to better document the physical and psychological health problems women subjected to IPV experience over time, and what types of social services and health care interventions are most timely and impactful. We reemphasize previous calls to increase IPV-related psychological abuse and brain injury awareness, screening, and interventions to improve cognitive functioning, resiliency, and well-being of women who experience IPV (Adhikari et al., 2024; Ogbe et al., 2020; Toccalino et al., 2024; Yastibas-Kaçar et al., 2024).

## Figures and Tables

**Table 1:** Sample construction process

	(*n*) Excluded	(*N*) Retained
Number of subjects who participated in the study	-	124
Subjects who did not meet the study’s inclusion criteria		
Living in shelter	2	122
Mild IPV-related BI in past 3 months	2	120
Mild non-IPV related BI in past 3 months	1	119
Moderate to severe IPV-related BI	7	112
Moderate to severe childhood BI	2	111^(a)^
Moderate to severe other BI	5	106
Convulsions/epilepsy	5	103^(b)^
Stroke	6	99^(c)^
Brain surgery	2	97
Final sample retained for regression analyses		97^(d)^

*IPV* = intimate partner violence, *BI* = brain injury

For^(a)–(c)^ the (n) is greater than what was dropped from (N) because subjects met multiple exclusion criteria and were already dropped in row(s) above: i.e.,

(a) Moderate-severe IPV-related BI and moderate-severe childhood BI

(b) Moderate-severe IPV-related BI and convulsions/epilepsy

(b) Moderate-severe other BI and convulsions/epilepsy

(c) Moderate-severe IPV-related BI, convulsions/epilepsy, and stroke

(c) Moderate-severe other BI and stroke

(d) Due to listwise deletion of missing observations across all study variables, the retained sample size drops from *N*=97 to *N*=70 to *N*=76 in different regressions

**Table 2:** Central nervous system vital signs tests

Domain Name	Test Name	What is Measured	Test Description
Reaction Time	Stroop Test	Processing speed and complex reaction time embedded in a Stroop inhibitory control test.	This task had three parts. First, participants pressed the spacebar when the words, RED, YELLOW, BLUE, and GREEN (printed in black) appeared on the screen. In the subsequent parts, the words were printed in color. In the second part, participants pressed the spacebar when the color of the word matched its meaning. Third, participants pressed the spacebar when the color of the word did not match its meaning. CNS-VS calculated the score by taking an average of correct responses in parts two and three.
Verbal Memory	Verbal Memory	Immediate and delayed recognition memory for a word list presented visually.	Participants saw fifteen words on a screen, one by one, every two seconds. In the immediate recognition trial, participants had to recognize those words among fifteen new words. After six other tests, there was a delayed recognition memory trial. Participants responded by pressing the spacebar. CNS-VS calculated the score as follows: immediate correct hits + immediate correct passes + delayed correct hits + delayed correct passes.
Visual Memory	Visual Memory	Immediate and delayed recognition memory for designs.	Participants saw fifteen geometric figures on a screen, one by one. In the immediate recognition trial, participants had to recognize those figures among fifteen new figures. After six other tests, there was a delayed recognition memory trial. Participants responded by pressing the spacebar. CNS-VS calculated the score as follows: immediate correct hits + immediate correct passes + delayed correct hits + delayed correct passes.
Processing Speed	Symbol Digit Coding	Visual scanning and processing speed.	Towards the top of the screen, there was a table with two rows: the top row had eight symbols, one of which was highlighted, and the bottom had numbers (2–9) corresponding to each symbol. Towards the bottom of the screen, participants were presented with a series of screens that each had a table with eight symbols on the top row and eight empty boxes below, where participants had to type the number that matched the highlighted symbol. CNS-VS calculated the score as follows: correct responses - errors.
Executive Function	Shifting Attention Test	Set-shifting and processing speed.	One figure was shown at the top of the screen (either a square or circle) and two figures on the bottom (square and circle). The figures were either red or blue, assigned randomly. Participants had to match one of the bottom figures to the top one based on either shape or color; the rules changed randomly. CNS-VS calculated the score as follows: correct responses - errors.

**Table 3:** Characteristics of the sample

	*N*	Range^(a)^	Mean or %^(a)^	SD	Skewness^(b)^	Kurtosis
Demographics						
Age (Years)	97	19–69	39.03	12.75	0.36	−0.86
Education (Years)	97	9–23	15.81	2.54	0.22	0.67
Currently employed (%)	97	--	65%	0.48	--	--
Race/Ethnicity (%)	97					
White	61	--	63%	0.49	--	--
Hispanic	18	--	19%	0.39	--	--
Black	9	--	9%	0.29	--	--
Asian	6	--	6%	0.24	--	--
Native American	1	--	1%	0.10	--	--
Hawaiian/Pacific Islander	1	--	1%	0.10	--	--
Multiracial	8	--	8%	0.28	--	--
Other	2	--	2%	0.14	--	--
Neuropsychological test scores						
Reaction Time	94	41–117	90.53	15.21	−0.73	0.33
Verbal Memory	93	42–126	94.70	18.14	−0.66	0.26
Visual Memory	93	56–129	96.86	15.32	−0.46	−0.15
Processing Speed	94	67–139	99.32	13.63	0.20	0.15
Executive Function	91	40–119	93.59	14.74	−0.91	1.48
Characteristics used as predictors						
Number of IPV-related mild BIs^(c)^	97	0–25	2.67	5.12	3.17	10.53
Psychological abuse severity (CTS2)	79	0–366	124.11	79.15	0.95	0.98
Characteristics used as covariates						
Number of non-IPV related mild BIs^(c)^	97	0–25	2.05	4.75	4.08	16.87
Number of non-IPV related mild BIs (log)	97	0–1.41	0.29	0.34	1.48	2.49
Partner negotiation (CTS2)	83	2–300	101.90	78.51	0.68	−0.51
Alcohol use in past year (AUDIT)^(c)^	96	0–38	3.36	5.89	4.25	20.84
Alcohol use in past year (log)	96	0–1.59	0.46	0.36	0.75	0.78
Chronic drug use history (%)	96	--	4%	0.20	--	--
Current psychotropic medication use (%)	96	--	52%	0.50	--	--
Childhood trauma (CTQ)	97	25–115	57.82	21.73	0.34	−0.61
PTSD (PCL-5)	97	1–75	31.58	18.51	0.37	−0.75
Depression (PHQ-9)	97	0–25	9.58	5.72	0.60	−0.05
Anxiety (GAD-7)	97	0–21	7.86	5.60	0.73	−0.32
Years since psychological abuse^(c)^	96	0–43.25	4.76	6.88	2.75	10.55
Years since psychological abuse (log)	96	0–1.65	0.53	0.44	0.39	−0.93
Years since physical abuse	97	0.15–43.25	7.96	8.42	1.83	3.73
Age (Years)	97	19–69	39.03	12.75	0.36	−0.86
Education (Years)	97	9–23	15.81	2.54	0.22	0.67
Currently employed (%)	97	--	65%	0.48	--	--
IPV-related BI characteristics	97					
0 IPV-related mild BIs (%)	38	--	39%	0.49	--	--
1 or more IPV-related mild BIs (%)	59	--	61%	0.49	--	--
Years since IPV-related mild BI^(d)^	59	1.31–36.64	9.95	8.00	1.22	1.10

*IPV* = intimate partner violence, *BI* = brain injury, *CTS2* = Revised Conflict Tactics Scale 2, *AUDIT* = Alcohol Use Disorders Identification Test, *CTQ* = Childhood Trauma Questionnaire, *PTSD* = Posttraumatic Stress Disorder, *PCL-5* = PTSD Checklist-5, *PHQ-9* = Patient Health Questionnaire-9, *GAD-7* = Generalized Anxiety Disorder-7

(a) Means and ranges provided for continuous variables. % provided for dichotomous variables

(b) Skewness and kurtosis not applicable for dichotomous variables

(c) Skewed variables were log transformed (i.e., number of IPV and non-IPV related mild BIs, alcohol use in the past year, and years since psychological abuse). Results remained the same with or without log transformation. Results presented with log transformed variables, except for the number of IPV-related mild BIs (a main independent variable of interest that was not log transformed in prior studies) to maintain interpretability and comparability

(d) Descriptives reported only for participants who had IPV-related mild BI (*n* = 59). In comparison, all participants (*n* = 97) experienced physical abuse. The correlation between the years since IPV-related mild BI and years since physical abuse was (r =.70, *p* <.01). As such, we chose to only use years since physical abuse as a covariate in the regressions because it had a larger sample size, and we chose not to use both to avoid multicollinearity

**Table 4:** Regression results

	Model-1:		Model-2:		Model-3:		Model-4:		Model-5:	
	Reaction Time		Verbal Memory		Visual Memory		Processing Speed		Executive Function	
	Beta	p	Beta	p	Beta	p	Beta	p	Beta	p
(Constant)	76.72	0.000	55.08	0.008	80.53	0.00	95.77	0.00	88.24	0.00
Main variables										
Number of IPV mild BIs	−0.30	0.410	−0.39	0.376	−0.83	**0.033**	−0.26	0.429	−0.02	0.957
Psychological abuse (CTS2)	0.07	**0.002**	0.18	0.058	−0.01	0.807	0.00	0.968	0.05	0.082
Psychological abuse squared	-	-	−0.001	**0.039**	-	-	-	-	-	-
Control variables										
Number of non-IPV mild BIs	−4.13	0.495	−5.12	0.491	2.78	0.663	−3.39	0.537	−9.51	0.168
Partner negotiation (CTS2)	0.06	0.021	0.04	0.197	0.02	0.390	0.02	0.434	0.02	0.395
Alcohol use in past year (AUDIT)	6.11	0.295	−2.31	0.749	−6.37	0.304	4.74	0.369	5.32	0.437
Chronic drug use history	−26.02	0.013	1.45	0.915	4.69	0.669	−19.07	0.043	−13.10	0.252
Current psychotropic medication use	7.41	0.059	7.27	0.133	3.18	0.439	7.22	0.043	10.77	0.017
Childhood trauma (CTQ)	−0.10	0.29	−0.05	0.699	0.02	0.858	−0.09	0.272	−0.26	0.019
PTSD (PCL-5)	−0.38	0.038	0.86	0.0003	0.24	0.223	−0.13	0.445	−0.15	0.483
Depression (PHQ-9)	0.65	0.257	−1.79	0.014	−0.50	0.412	−0.49	0.347	−0.15	0.826
Anxiety (GAD-7)	0.29	0.562	−0.70	0.259	−0.40	0.455	0.54	0.232	0.89	0.111
Years since psychological abuse	−1.52	0.789	1.28	0.853	−5.41	0.369	−4.30	0.402	5.56	0.382
Years since physical abuse	0.13	0.723	−0.37	0.427	0.49	0.222	−0.19	0.588	−0.01	0.975
Age	0.13	0.505	0.07	0.755	−0.10	0.614	0.35	0.043	0.37	0.091
Years of education	−0.13	0.858	0.81	0.381	1.06	0.189	−0.18	0.79	−0.76	0.374
Currently employed	1.78	0.650	13.90	0.006	6.20	0.145	0.21	0.953	2.37	0.598
Model statistics										
N (observations)	73.00		74.00		74.00		73.00		70.00	
R-square	0.41		0.40		0.26		0.24		0.29	
Adjusted R-square	0.24		0.21		0.05		0.02		0.076	

*IPV* = intimate partner violence, *BI* = brain injury, *CTS2* = Revised Conflict Tactics Scale 2, *AUDIT* = Alcohol Use Disorders Identification Test, *CTQ* = Childhood Trauma Questionnaire, *PTSD* = Posttraumatic Stress Disorder, *PCL-5* = PTSD Checklist-5, *PHQ-9* = Patient Health Questionnaire-9, *GAD-7* = Generalized Anxiety Disorder-7. A visual inspection of the scatterplot and residual plot for Verbal Memory and psychological abuse severity showed that the data did not follow a linear pattern. A quadratic regression provided a better fit for the data. Hence, a psychological abuse severity squared term was included in the Verbal Memory model. *p* <.05 are highlighted in bold for main variables

## Data Availability

The data supporting this study are available from the corresponding author upon reasonable request. All data will be uploaded to Federal Interagency Traumatic Brain Injury Research (FITBR) data repository after completion of the study. https://fitbir.nih.gov/.
